# Oral Polio Vaccine Influences the Immune Response to BCG Vaccination. A Natural Experiment

**DOI:** 10.1371/journal.pone.0010328

**Published:** 2010-05-21

**Authors:** Erliyani Sartono, Ida M. Lisse, Elisabeth M. Terveer, Paula J. M. van de Sande, Hilton Whittle, Ane B. Fisker, Adam Roth, Peter Aaby, Maria Yazdanbakhsh, Christine S. Benn

**Affiliations:** 1 Department of Parasitology, Leiden University Medical Center, Leiden, the Netherlands; 2 Department of Pathology, Herlev University Hospital, Herlev, Denmark; 3 MRC Laboratories, Fajara, Gambia; 4 Bandim Health Project, Statens Serum Institut, Copenhagen, Denmark; 5 Department of Medical Microbiology, Lund University, Lund, Sweden; 6 Bandim Health Project, Indepth Network, Bissau, Guinea-Bissau; Singapore Immunology Network, Singapore

## Abstract

**Background:**

Oral polio vaccine (OPV) is recommended to be given at birth together with BCG vaccine. While we were conducting two trials including low-birth-weight (LBW) and normal-birth-weight (NBW) infants in Guinea-Bissau, OPV was not available during some periods and therefore some infants did not receive OPV at birth, but only BCG. We investigated the effect of OPV given simultaneously with BCG at birth on the immune response to BCG vaccine.

**Methods and Findings:**

We compared the in vitro and the in vivo response to PPD in the infants who received OPV and BCG with that of infants who received BCG only. At age 6 weeks, the *in vitro* cytokine response to purified protein derivate (PPD) of *M. Tuberculosis* was reduced in LBW and NBW infants who had received OPV with BCG. In a pooled analysis receiving OPV with BCG at birth was associated with significantly lower IL-13 (p = 0.041) and IFN-γ (p = 0.004) and a tendency for lower IL-10 (p = 0.054) in response to PPD. Furthermore, OPV was associated with reduced *in vivo* response to PPD at age 2 months, the prevalence ratio (PR) of having a PPD reaction being 0.75 (0.58–0.98), p = 0.033, and with a tendency for reduced likelihood of having a BCG scar (0.95 (0.91–1.00), p = 0.057)). Among children with a scar, OPV was associated with reduced scar size, the regression coefficient being −0.24 (−0.43—0.05), p = 0.012.

**Conclusions:**

This study is the first to address the consequences for the immune response to BCG of simultaneous administration with OPV. Worryingly, the results indicate that the common practice in low-income countries of administering OPV together with BCG at birth may down-regulate the response to BCG vaccine.

## Introduction

WHO recommends BCG vaccine and oral polio vaccine (OPV) at birth in low-income countries. BCG vaccine is the world's most widely used vaccine and although its efficacy is variable, it is still considered the best way to protect against childhood tuberculosis [1], [2]. BCG vaccination induces potent Th1 responses to mycobacterial antigen in newborns [3]. Studies in Gambia have shown that BCG vaccination affects responses to unrelated vaccines by enhancing the production of Th1 and Th2 type cytokines [3], [4]. BCG vaccination in early life also increased the antibody response to OPV [4]. The effect of providing OPV simultaneously with BCG on the immune response to BCG has never been studied.

We recently conducted two trials among newborns in Guinea-Bissau. According to local practice, low-birth-weight (LBW, <2500 g) infants only receive OPV at birth; BCG vaccine is usually postponed until the child reaches 6 weeks of age. In trial 1, LBW infants were randomized to receive an early BCG vaccine or the usual delayed BCG vaccine. In trial 2, normal birth-weight (NBW, ≥2500 g) children were randomized to vitamin A supplementation or placebo together with BCG vaccine [5]. The children were followed for mortality. Subgroups of the children were also followed for the *in vivo* and *in vitro* immune response to BCG [6]. OPV is recommended to be given at birth but for certain periods in 2004, during the conduct of the two trials, OPV was not available in Guinea-Bissau and hence some trial participants did not receive OPV at birth. This ‘natural experiment’ allowed us to compare in an observational, but rather unbiased manner, the effect of OPV provided with BCG at birth. Intriguingly, we observed that not receiving OPV at birth was associated with significantly decreased male mortality during the first year of life [7]. In the present study we investigated the effect of OPV given simultaneously with BCG at birth on the immune response to BCG vaccine.

## Materials and Methods

### Ethics Statement

This study was conducted according to the principles expressed in the Declaration of Helsinki. The study was approved by the research committee of the Ministry of Health in Guinea-Bissau and for European Centers by the Danish Central Ethical Committee. All mothers provided written informed consent for the collection of samples of their children.

### Setting

The two large randomized trials were conducted by the Bandim Health Project (BHP) in Bissau, Guinea-Bissau. The BHP covers a population of around 100,000 in 6 districts in the urban area by a demographic surveillance system. All houses are visited every month to register new pregnancies and births. Once a newborn is identified, the child is followed with a home visit every third month. There is a national hospital (NH) with a maternity ward in the capital and three health centers in the study area, one of them with a maternity ward. Around 60–70% of the mothers from the study area deliver at the NH or at the health center maternity ward. Since 2002 all LBW infants born at the NH have been followed for one year, also infants outside the study area. In trial 1, all LBW infants born at the NH or in the study area were invited to participate in a trial studying the effect of receiving early BCG vaccination on mortality and cytokine levels in LBW infants. In trial 2, NBW infants from the study area were invited to participate in a trial studying the effect of vitamin A supplementation at birth on mortality and cytokine levels. These two EU funded trials were carried out within a collaborative network of European and Guinea-Bissau institutions. The trials were registered at www.clinicaltrials.gov, registration number, NCT00146302 and NCT00168597.

### Enrolment into the trials

**Figure 1:**
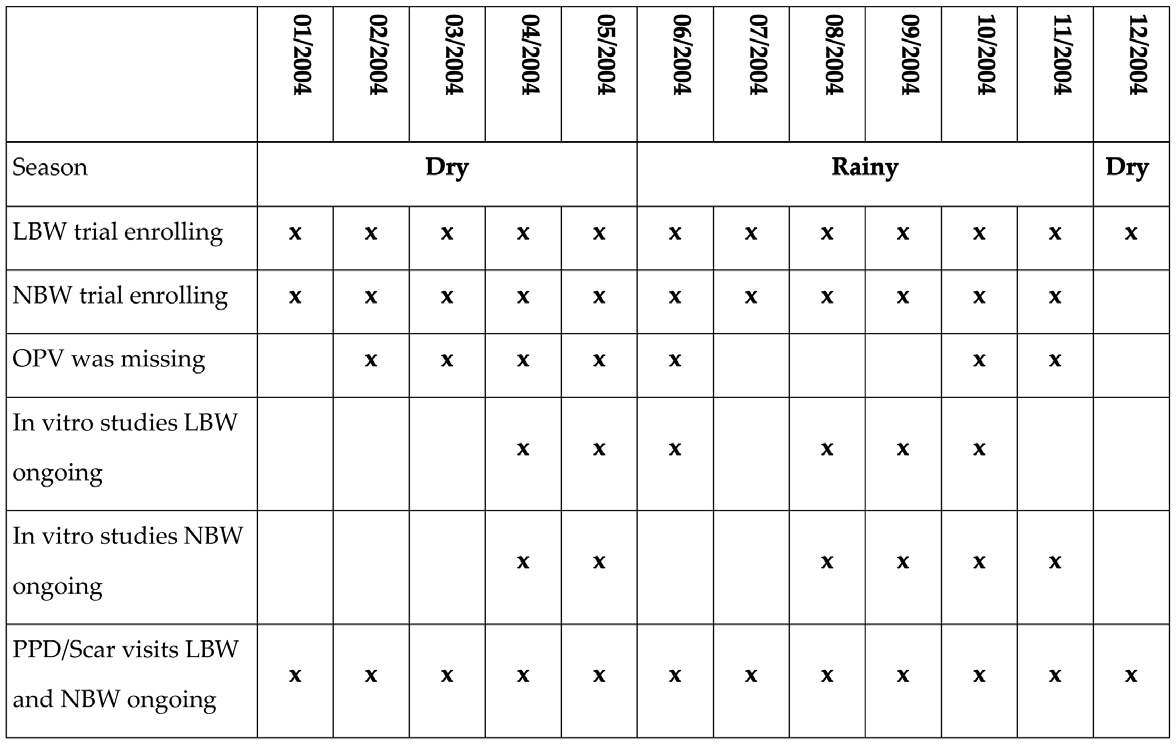
An overview of the two trials, the periods with missing OPV, and the periods with ongoing subgroup studies in Guinea-Bissau 2004.

Newborns were enrolled throughout 2004 (Figure 1). Mothers were informed of the purpose of the study. If a mother of a LBW infant accepted to participate, she drew a randomization number indicating whether the children would be BCG vaccinated early or not. If a mother of a NBW infant accepted to participate, she drew a randomization number indicating whether the children would receive vitamin A supplementation (50,000 IU) or placebo. At the time of enrolment, children were examined clinically. The infants were weighed and length, head-circumference and mid-upper-arm-circumference (MUAC) were measured. Newborns that were overtly ill were not enrolled but referred to treatment.

LBW infants who were randomized to not receiving early BCG vaccination were not included in the present study since we aimed to look at the effect of OPV on the immune response to BCG given at the same time. Hence, the LBW population only includes children who were randomized to BCG at birth. NBW children who had received vitamin A supplementation at birth were not included in the present study as this could have affected their response to OPV and BCG. Hence, the NBW population only includes children who were randomized to placebo at birth.

### Vaccines

BCG (0.05 ml; Statens Serum Institut, Copenhagen, Denmark) was provided by the BHP and given intradermally in the upper left deltoid region. OPV was supplied through the national immunization programme and given orally. Three types of OPV were used in Guinea-Bissau, produced by Novartis, Sanofi Pasteur, and GlaxoSmithKline. However, OPV was missing during periods of 2004. As part of our trial routines, we noted at the inclusion form whether the children received OPV or not. Based on the distribution of children who had not received OPV, OPV was missing at one or more inclusion sites 1) from the beginning of February 2004 to the beginning of June 2004, 2) again in the end of June 2004, and 3) from the end of October 2004 to November 2004 (Figure 1).

### Follow-up for *in vitro PPD response*

We aimed to enroll 200 children from trial 1 and 400 children from trial 2 in a subgroup study of cytokine production. The children were enrolled between April and November 2004 (Figure 1). We attempted to visit the children at 6 weeks of age. A blood sample was collected by finger prick and analyzed for *in vitro* cytokine responses. Whole blood cultures were done as previously described [6], [8]. Briefly, heparinized finger prick blood was diluted in RPMI 1640 medium (Invitrogen, Breda, The Netherlands) to a final concentration of 1 in 10 and culture in a total volume of 200 µl in 96-well U plate (Nunc, Roskilde, Denmark) with the presence of purified protein derivative of *Mycobacterium tuberculosis* (PPD, 10 µg/ml; Statens Serum Institut, Denmark, Copenhagen). Supernatants were collected on day 3 and stored at −80°C.

### Measurement of cytokine levels

IL-5, IL-10, IL-13, IFN-γ and TNF-α from day 3 supernatants were analyzed simultaneously using commercial Luminex cytokine kit and buffer reagent kit (BioSource, Camarillo, CA, USA) and run on a Luminex-100 cytometer (Luminex Corporation, Austin, TX, USA), equipped with StarStation software (Applied Cytometry Systems, Dinnington, UK). The assay was performed with slight modification from the manufacturer's recommendation. Briefly, assays were done in a 96-well U plate at room temperature. Mixes of beads were incubated for 2 hours with a standard, samples, or blank in a final volume of 50 µl for 2 hours under continuous shaking. Beads were washed twice and incubated with cocktail of biotinylated antibodies (25 µl/well) for 1 hour. After removal of excess biotinylated antibodies, Streptavidin-PE was added for 30 minutes. The plate was then washed and analyzed. The lower detection limit of the assays was 3 pg/ml, 5 pg/ml, 10 pg/ml, 5 pg/ml and 10 pg/ml for IL-5, IL-10, IL-13, IFN-γ and TNF-α, respectively. Samples with concentrations below the detection limit were given the value of this threshold.

### Follow-up for BCG scar and *in vivo* PPD response

All LBW children were visited at home and examined for BCG scar and *in vivo* PPD response at 2 and 6 months of age. In four of six study area districts NBW children were examined for BCG scar and *in vivo* PPD response when they reached 2 and 6 months of age. At the home visits a trained nurse documented health status and vaccination status. She measured the size of the scar following BCG vaccination. Subsequently, 0.1 ml of Tuberculin PPD RT23 (Statens Serum Institut, Copenhagen, Denmark) was injected intradermally on the ventral side of the forearm. Between 48 and 72 hours later, the child was visited again, and the induration was measured by a trained field worker using track-ball technique [9]. Size of scar and induration was defined as the average of the height and the width measured to nearest millimeter with a transparent ruler. Children with a measurable scar were categorized as “scar-positive”. Children with a mean diameter of the induration ≥2 mm were categorized as “PPD-positive”. Children who had taken chloroquine within seven days prior to the visit were excluded from the PPD analysis, since chloroquine treatment is known to suppress PPD reaction [10]. These children were retained in the analysis of scar reaction since we considered it unlikely that current chloroquine treatment would have affected the scar formation. Children who had received BCG vaccination less than 30 days prior to the visit or with known exposure to tuberculosis in the household within the previous 6 months (information only available for NBW population) were excluded. Children with PPD reactions >10 mm were referred to a medical doctor to be further examined for tuberculosis (TB).

### Statistical analysis

All statistical analysis was performed in Stata 10.0.

#### *In vitro* PPD response

**Figure 2:**
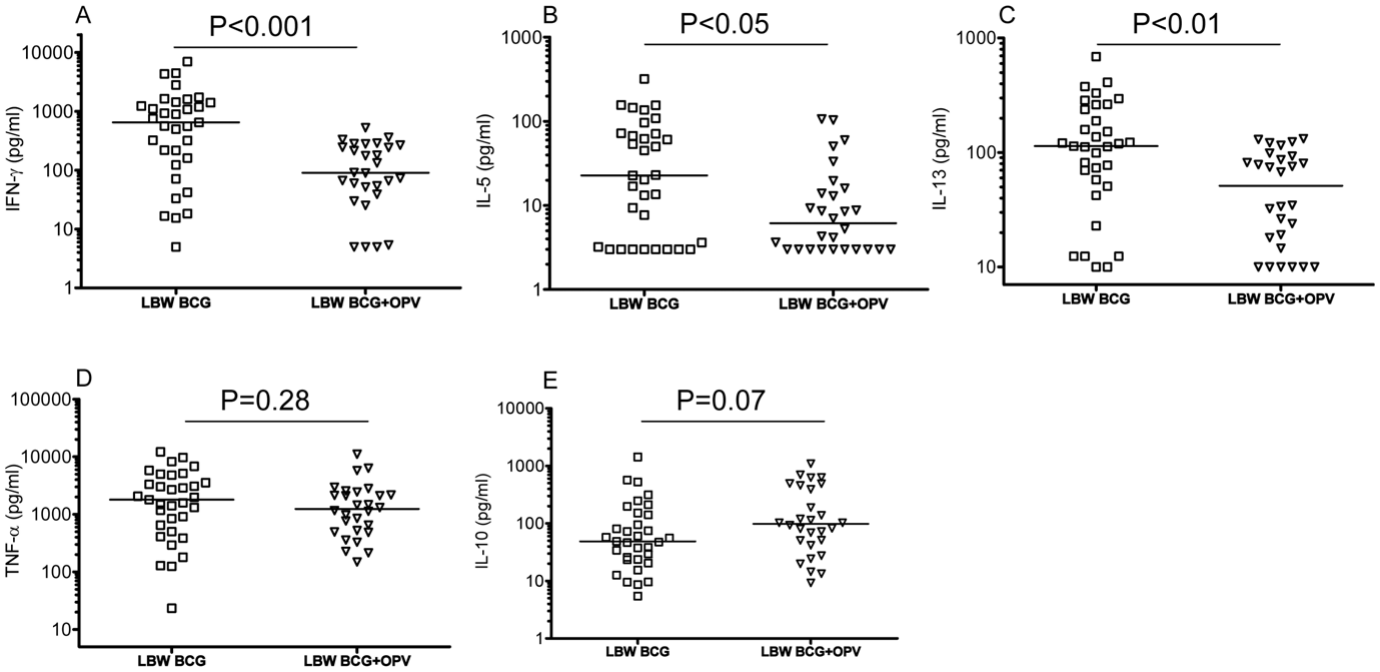
PPD stimulated IFN-γ (A), IL-5 (B), IL-13 (C), TNF-α (D) and IL-10 (E) production in low birth weight BCG (LBW BCG) and BCG+OPV vaccinated (LBW BCG+OPV) infants. One dot represents one individual. Horizontal lines represent medians.

**Figure 3:**
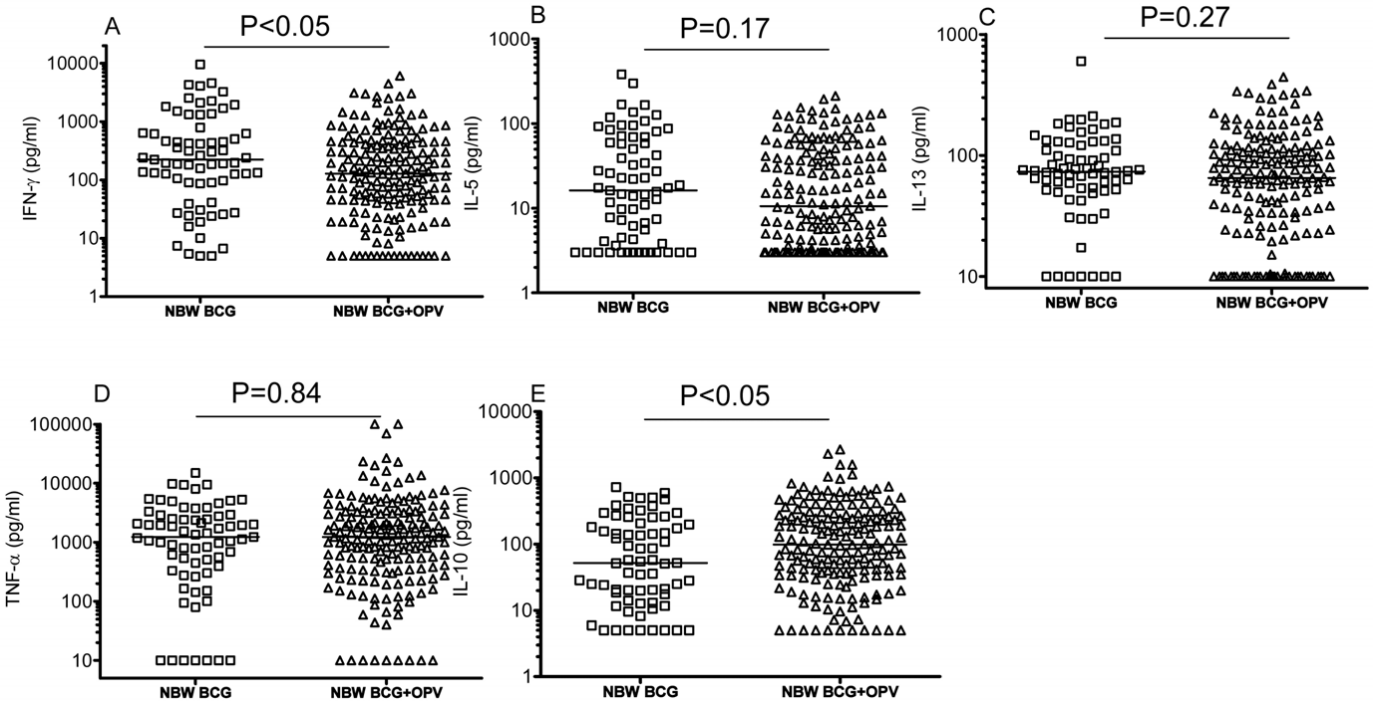
PPD stimulated IFN-γ (A), IL-5 (B), IL-13 (C), TNF-α (D) and IL-10 (E) production in normal birth weight BCG (NBW BCG) and BCG+OPV vaccinated (NBW BCG+OPV) infants. One dot represents one individual. Horizontal lines represent medians.

Cytokine levels were not normally distributed. The crude cytokine levels were first compared in a univariate analysis using non-parametric Mann-Whitney *U* tests, and the results were provided in the figures of the distribution of cytokine levels in the different groups (Figures 2 and 3). Subsequently, the frequency of low and high cytokine producers in BCG and BCG+OPV recipients was compared in Poisson regression model with robust variance estimates [11] using the median cytokine level in the total population as cut off for low and high responder. Thus, the relative measure is a prevalence ratio (PR). The study population was divided into 4 groups according to the birth weight and whether OPV was given together with BCG at birth (Table 1). As the periods with lack of OPV fell primarily in the dry season (from December to May), all children who had received BCG+OPV had been vaccinated in the rainy season (June to November). The two LBW groups were similar with respect to age and sex. However, MUAC at enrolment was larger in the BCG+OPV group compared with the BCG group, and they had received more OPV and more diphtheria-tetanus-pertussis (DTP) vaccines (recommended at age 6, 10 and 14 weeks) between enrolment and blood sampling (Table 1). The two NBW groups were similar with respect to age and MUAC at enrolment (Table 1). However, they were significantly different with regard to sex, the NBW BCG+OPV group having fewer boys. The two groups also differed significantly with regard to vaccinations received between enrolment and blood sampling (more OPV and less DTP in the BCG+OPV group). All further analyses were adjusted for age, sex, month of enrolment, MUAC at enrolment, and vaccinations between enrolment and blood sampling.

**Table 1:** Description of the cytokine study population.

Group	N (boys/girls)	Vaccinated in rainy season	Median MUAC at BCG vaccination (10–90 centiles)	Received OPV between vaccination and blood sample	Received DTP between vaccination and blood sample	Median age at sampling (10–90 centiles)
**Low-birth-weight infants**						
BCG only	33 (11/22)	15 (45%)	82 mm (70–88)	3 (9%)	2 (6%)	44 days (38–49)
BCG+OPV	28 (8/20)	28 (100%)	82 mm (70–94)	17 (61%)	7 (25%)	48 days (34–56)
**Normal-birth-weight infants**						
BCG only	69 (44/25)	12 (17%)	100 mm (88–112)	24 (35%)	20 (29%)	43 days (37–59)
BCG+OPV	179 (77/102)	179 (100%)	100 mm (88–108)	122 (68%)	26 (15%)	42 days (36–54)

#### BCG scar and *in vivo* PPD response

PPD response (yes/no) and scar response (yes/no) were analyzed as prevalence data using a Poisson regression model with robust variance estimates providing prevalence ratios (PR) [11]. Children with a PPD response above 15 mm were excluded from the analyses as they had potentially been exposed to TB. There were no such children among LBW children. Among NBW children one boy (BCG+OPV) had a PPD response above 15 mm at age 2 months and was excluded from further analyses. At age 6 months, one boy (BCG+OPV) and one girl (BCG), had a PPD response above 15 mm and were excluded from the 6 month-analysis. As in the cytokine analysis, there was a clear overweight of children who had received BCG+OPV in the rainy season, though there was more variation because the follow-up for PPD/scar had been conducted throughout 2004 (Figure 1). Among LBW infants, the BCG+OPV recipients only differed from the BCG only recipients with regard to season (Table 2). Among NBW infants, the two groups also differed with regard to MUAC at enrolment, and number of OPV received between enrolment and the PPD/Scar assessment (Table 2). We adjusted the analyses for sex, season (the larger variation in this analysis compared with the cytokine analysis allowed us to control for season rather than month), MUAC, and subsequent vaccinations. As in previous studies of PPD responses, the reading of the PPD reaction differed significantly by the assistant doing the reading and the analyses of PPD responses were therefore furthermore adjusted for assistant. Among PPD responders and/or scar reactors, we analyzed the size of the reaction in linear regression models controlling for the same potential confounders.

**Table 2:** Description of the scar and PPD study population.

Group	N (boys/girls)	Vaccinated in rainy season	Median MUAC at vaccination (10–90 centiles)	Received OPV between vaccination and PPD assessment	Received DTP between vaccination and PPD assessment	Median age at sampling (10–90 centiles)
**Low-birth-weight infants**						
BCG only	146 (57/89)	49 (34%)	82 mm (70–88)	61 (42%)	103 (71%)	65 days (57–79)
BCG+OPV	104 (43/61)	66 (63%)	82 mm (70–90)	47 (45%)	74 (71%)	64 days (59–75)
**Normal-birth-weight infants**						
BCG only	253 (143/110)	74 (29%)	96 mm (88–110)	135 (53%)	230 (91%)	63 days (54–82)
BCG+OPV	287 (146/141)	228 (79%)	100 mm (88–112)	184 (64%)	257 (90%)	64 days (55–78)

## Results

### *In vitro* PPD response

This analysis included 61 LBW infants who had been randomized to early BCG; 33 who had not received OPV at birth together with BCG and 28 who had. Furthermore, 248 NBW infants who had been randomized to placebo; 69 who had not received OPV and 179 who had were included.

### Influence of OPV on cytokine responses to PPD in LBW infants

When OPV was given together with BCG, there was a significant down regulation of IL-5, IL-13, and IFN-γ in response to PPD as compared to the group of infants who had received BCG only (Figure 2)(Table 3). IL-10 to PPD was higher in LBW infants who had received BCG together with OPV in the crude analysis, but the difference disappeared in the adjusted analysis (Table 3). There were no significant differences in TNF-α to PPD between the two groups (Figure 2)(Table 3).

**Table 3:** Comparison of the frequency of cytokine responders in children vaccinated with BCG and with BCG+OPV.

Response to PPD	Proportion of high responders (%)		Prevalence Ratio (95% CI) BCG+OPV versus BCG only	Adjusted Prevalence Ratio (95% CI) BCG+OPV versus BCG only
	BCG+OPV	BCG only		
**Low-birth-weight infants**				
	(N = 28)	(N = 33)		
IL-5	9 (32%)	21 (64%)	**0.51 (0.28–0.92)**	0.56 (0.28–1.12)
IL-10	17 (61%)	11 (33%)	**1.82 (1.03–3.23)**	0.46 (0.14–1.49)
IL-13	13 (46%)	23 (70%)	0.67 (0.42–1.06)	0.51 (0.22–1.16)
IFN-γ	12 (43%)	24 (73%)	**0.59 (0.36–0.95)**	0.40 (0.15–1.09)
TNF-α	13 (46%)	20 (61%)	0.77 (0.47–1.25)	2.25 (0.74–6.85)
**Normal birth-weight infants**				
	(N = 179)	(N = 69)		
IL-5	83 (46%)	46 (58%)	0.80 (0.62–1.03)	0.73 (0.42–1.26)
IL-10	96 (54%)	31 (45%)	1.19 (0.89–1.60)	0.63 (0.32–1.21)
IL-13	82 (46%)	36 (52%)	0.88 (0.67–1.16)	0.66 (0.38–1.15)
IFN-γ	78 (44%)	41 (59%)	**0.73 (0.57–0.95)**	**0.54 (0.30–0.97)**
TNF-α	87 (49%)	34 (49%)	0.99 (0.74–1.31)	0.88 (0.40–1.95)

Adjusted for age, sex, MUAC and month of enrolment, subsequent vaccinations.

### Influence of OPV on cytokine responses to PPD in NBW infants

In the unadjusted analysis IFN-γ responses to PPD appeared depressed and IL-10 responses increased when OPV was given with BCG (Figure 3). In the adjusted analysis, the IFN-γ levels to PPD were significantly lower in BCG+OPV compared with BCG only vaccinated infants and there was a similar tendency for IL-13 (Table 3).

### Influence of OPV on cytokine responses to PPD in all infants

In a pooled analysis of LBW and NBW infants, receiving OPV with BCG at birth was associated with significantly lower IL-13 and IFN-γ in responses to PPD; the adjusted PRs (furthermore adjusted for being LBW) were 0.64 (0.41–0.98)(p = 0.041), and 0.51 (0.33–0.80)(p = 0.004), respectively. The IL-10 response to PPD tended to be lower as well (0.61 (0.36–1.01), p = 0.054).

### BCG scar and *in vivo* PPD response

Among LBW infants enrolled in 2004, 250 BCG recipients were visited; 104 had received BCG+OPV, 146 BCG only. We obtained a scar reading in 231 children at age 2 months and 200 children at age 6 months and a valid PPD assessment of 202 at age 2 months, and 170 at age 6 months. Among NBW infants enrolled in 2004, 540 placebo recipients had their BCG scar and PPD response assessed once or more; 287 had received BCG+OPV; 253 BCG only. We obtained a scar reading in 438 children at age 2 months and 428 children at age 6 months and a valid PPD assessment of 379 at age 2 months, and 327 at age 6 months.

#### Influence of OPV on PPD/Scar responses in LBW infants

At *age 2 months*, receiving OPV with BCG at birth was associated with a borderline significantly lower likelihood of being PPD positive (Table 4). There was no association with size of the reaction among the few children who had a positive reaction. Almost all children were scar positive, and there was no association between OPV at birth and being scar positive (Table 5), or the size of the scar among scar positives (data not shown).

**Table 4:** The effect of receiving OPV with BCG at birth on the likelihood of having an *in vivo* response to PPD at age 2 months and 6 months.

	ALL CHILDREN	CRUDE PR (95% CI) BCG+OPV VERSUS BCG ONLY	ADJUSTED PR (95% CI) BCG+OPV VERSUS BCG ONLY
	Positive/all (%)		
**Low-birth-weight infants**			
2 months			
BCG+OPV	7/87 (8%)	0.49 (0.21–1.11)	0.43 (0.18–1.02)**
BCG only	19/115 (17%)		
6 months			
BCG+OPV	17/71 (24%)	0.85 (0.50–1.43)	0.65 (0.39–1.13)
BCG only	28/99 (28%)		
**Normal-birth-weight infants**			
2 months			
BCG+OPV	70/208 (34%)	0.81 (0.62–1.05)	0.83 (0.62–1.09)
BCG only	71/170 (42%)		
6 months			
BCG+OPV	56/167 (34%)	1.01 (0.74–1.38)	1.09 (0.75–1.59)
BCG only	52/157 (33%)		

Adjusted for sex, MUAC and season of enrolment, subsequent vaccinations and assistant.

**P = 0.056.

**Table 5:** The effect of receiving OPV with BCG at birth on the likelihood of being scar positive at age 2 and 6 months.

	ALL CHILDREN	PR (95% CI) BCG+OPV VERSUS BCG ONLY	ADJUSTED PR (95% CI) BCG+OPV VERSUS BCG ONLY
	Positive/all (%)		
**Low-birth-weight infants**			
2 months			
BCG+OPV	90/97 (93%)	1.00 (0.93–1.08)	1.00 (0.92–1.08)
BCG only	124/134 (93%)		
6 months			
BCG+OPV	74/78 (95%)	0.98 (0.92–1.04)	0.98 (0.92–1.05)
BCG only	118/122 (97%)		
**Normal-birth-weight infants**			
2 months			
BCG+OPV	203/227 (89%)	0.95 (0.90–1.00)	**0.93 (0.87–0.99)** **
BCG only	198/210 (94%)		
6 months			
BCG+OPV	217/227 (96%)	0.98 (0.95–1.02)	0.99 (0.95–1.04)
BCG only	193/198 (97%)		

Adjusted for sex, MUAC and season of enrolment, and subsequent vaccinations.

**P = 0.015.

At *age 6 months*, the lower likelihood of being PPD positive (Table 4) was no longer significant for the OPV group and there was no association with size of the reaction among the few children who had a positive reaction. Almost all children were scar positive, and there was no association between OPV at birth and being scar positive (Table 5), or the size of the scar among scar positives (data not shown).

#### Influence of OPV on PPD/Scar responses in NBW infants

At *age 2 months*, receiving OPV with BCG at birth was not associated with the likelihood of being PPD positive (Table 4), nor was it associated with the size of the response among children with a positive response (data not shown). Adjusted for potential confounders, children who had received OPV at birth had a lower likelihood of developing a BCG scar (Table 5). Receiving OPV at birth was negatively associated with the size of the scar among those who had a scar (regression coefficient −0.36 (−0.57—0.15); p = 0.001).

At *age 6 months*, receiving OPV with BCG at birth was not associated with the likelihood of being PPD positive (Table 4) or the size of the PPD reaction (data not shown). Almost all children had a scar at age 6 months and receiving OPV at birth was not associated with the likelihood of being scar positive (Table 5) or the size of the scar (data not shown).

#### Influence of OPV on PPD/Scar responses in all infants

**Figure 4:**
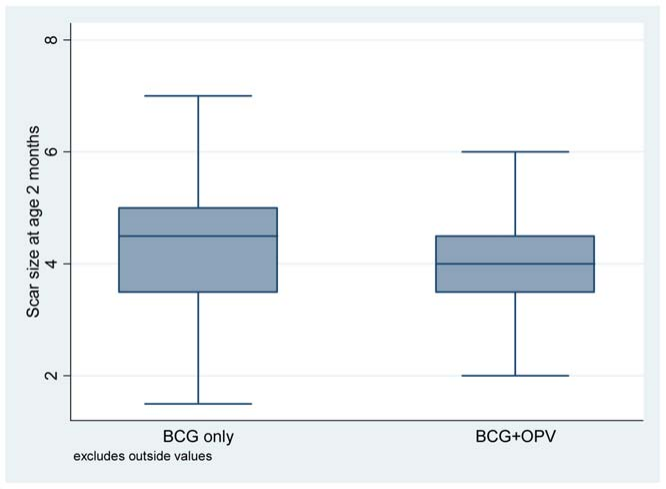
Comparison of BCG scar size at age 2 months between infants who got or did not get OPV with BCG at birth.

In a pooled analysis of LBW and NBW infants, receiving OPV with BCG at birth was associated with significantly lower likelihood of being PPD positive at age 2 months, the PR being 0.75 (0.58–0.98), p = 0.033. OPV with BCG was not associated with size of the PPD reaction (data not shown). OPV with BCG was also tended to be associated with a reduced likelihood of being scar positive (0.95 (0.91–1.00), p = 0.057). Among scar positive children, OPV was associated with significantly reduced scar size, the regression coefficient being −0.24 (−0.43—0.05), p = 0.012 (Figure 4). No significant differences were seen at age 6 months.

## Discussion

This study is the first to demonstrate that mucosal administration of OPV simultaneously with intradermal BCG vaccination may have profound immunological consequences. By comparing immune responses of infants who had received BCG at birth either with or without OPV, we observed that OPV had a significant suppressing effect on Th1 and Th2 responses to PPD. These *in vitro* findings were paralleled by findings of significantly reduced *in vivo* responses to PPD, a tendency for less BCG scars, and significantly reduced BCG scar size among those who developed a scar.

### Strengths and weaknesses

Though OPV is widely used, little is known regarding the cellular immune responses following vaccination, in particular its non-specific immune responses to un-related stimuli. The effect of OPV could be studied in the present study due to a temporary shortage of the vaccine in Guinea-Bissau during several periods of 2004. The shortage occurred in a period when two trials, examining among other things the immune responses of newborns, were being conducted. It was therefore possible to study in a relatively unbiased manner the effect of OPV on the *in vitro* response to PPD, as well as the effect on two markers of BCG immunity: PPD responses and BCG scarification.

Strengths of our study include the design; it was mainly logistic factors which determined whether the children would receive OPV or not. Despite this, there were some clear differences between the children who did or did not receive OPV at birth for instance with regard to season of vaccination, anthropometrics at inclusion, and other vaccines received. We conducted our analyses with and without adjustment for these co-variables, and indeed noted that the crude estimated changed considerably when we included the other variables as evidence of confounding. We cannot exclude that there is considerable residual confounding due to for instance the burden of maternal infectious diseases, other vaccines received or nutritional status. Especially, residual confounding by season could be a problem. A recent study in Malawi found that *in vitro* IFN-γ response to PPD varied strongly by season, being lowest in the warm and rainy season as compared with the cool and dry season and the hot and dry season [12]. In Guinea-Bissau there are two distinct seasons. Our periods without OPV coincided with the dry season. Hence, we have largely compared children who received OPV in the rainy season with children who received no OPV in the dry season. If the IFN-γ response to PPD was depressed for other reasons in the rainy season it could be at least part of the reason for the observed lower IFN-γ response to PPD response in OPV recipients. However, we found the same results when we limited our analysis to the rainy season, though the confidence intervals of course became wider (data not shown). Furthermore, we found depressed *in vivo* responses to PPD as well. The PPD data set was larger than the cytokine data set, and had more seasonal variation and thereby more power to control adequately for season. The validity of our findings is supported by the fact that the findings from the LBW and NBW cohorts consistently pointed in the same direction, and the pooled analyses provided significant results. Nonetheless, our findings should be interpreted with caution.

Previously, it has been shown that BCG vaccination given at birth in either NBW or LBW infants enhanced immune response to PPD, characterized by elevated IL-5, IL-13 and IFN-γ levels (4, and unpublished data). In the present study, the administration of OPV at the time of BCG priming at birth, reduced Th1 and Th2 cytokine responses to mycobacterial antigens in LBW infants, suggesting that OPV down regulated cellular immune responses to BCG vaccine when the vaccines were administrated at the same time.

One possible mechanism whereby OPV co administered with BCG leads to a reduced cellular response to PPD may be competition of OPV and BCG activated T cells for resources and space [13], [14]. A second possible mechanism is that polio virus may have specific immune modulatory molecules that down regulate immune responses to antigens to which immune responses are being mounted simultaneously [15].

The fact that the downregulation of cytokine production to PPD as a result of OPV vaccination was more profound in LBW compared to NBW infants might be due to functional immaturity of LBW infants. A recent study by Klein et al. showed that after inactivated polio vaccine, preterm infants had comparable frequencies of poliovirus specific CD4^+^CD45RO^+^CD69^+^IFN-γ memory T cells but diminished lymphoproliferation compared to term infants [16]. Furthermore, it is also possible the differences between LBW and NBW infants were caused by differences in the genetic make up. A study by Anuradha et al. reported that IFN-γ low producer genotype +874T/A were over represented in BCG non-responding children [17].

### Implications for BCG immunity

There is considerable evidence, both in humans and animals that IFN-γ production is necessary for protective immunity against mycobacterial disease [18]–[21] and successful chemotherapy of TB is associated with augmented IFN-γ production [22]–[24]. Furthermore, it has also been reported that individuals with genetic abnormalities of the IFN-γ receptor are highly susceptible to intracellular infections [25]–[27]. Thus, immunity to tuberculosis might be impaired by the down regulation of this Th1 response in LBW children receiving OPV at the same time as BCG at birth. The finding is supported by the fact that we found reduced PPD responses and reduced BCG scarification and scar size at age 2 months although these are debatable markers for TB immunity. Interestingly, the differences were no longer significant at age 6 months. It could be speculated that the PPD skin test at 2 months of age boosted the immune response to BCG and this obliterated the differences at 6 months. It has previously been reported that PPD skin test boosts the subsequent skin test reactivity [28]. The clinical implications of this early depression in immune responses to BCG need to be addressed in well-designed randomized trials with sufficient follow-up.

It is worth noticing that the protection induced by BCG against tuberculosis has been found to be much higher in high-income countries than in low-income countries. High-income countries mainly administer inactivated polio vaccine (IPV) at several months of age. Though many of the BCG-efficacy studies were done many years ago, before the introduction of OPV at birth, it may be speculated that the addition of OPV to BCG at birth has further compromised the protection induced by BCG in low-income countries.

### Conclusions

In summary, we are aware that the study population is small, and though we were able to control for a number of confounders there may be residual confounding. However, the potential effect of OPV on the immune response to BCG vaccine has not been studied before, and it may seem biologically plausible that the two vaccines interact with each other. The potentially negative effect of OPV on the immune response to BCG vaccine should be tested in a randomized trial.
